# Oligodendrocytes, BK channels and the preservation of myelin

**DOI:** 10.12688/f1000research.53422.2

**Published:** 2021-11-15

**Authors:** Maddalena Rupnik, David Baker, David L. Selwood

**Affiliations:** 1 Wolfson Insitute for Biomedical Research, University College London, London, WC1E 6BT, UK; 2 Centre for Neuroscience and Trauma, Blizard Institute, Queen Mary University of London, London, WC1E 6BT, UK

**Keywords:** KCNMA1, KCNMB4, big conductance Ca2+ activated K+ channel, oligodendrocytes, remyelination

## Abstract

Oligodendrocytes wrap multiple lamellae of their membrane, myelin, around axons of the central nervous system (CNS), to improve impulse conduction. Myelin synthesis is specialised and dynamic, responsive to local neuronal excitation. Subtle pathological insults are sufficient to cause significant neuronal metabolic impairment, so myelin preservation is necessary to safeguard neural networks. Multiple sclerosis (MS) is the most prevalent demyelinating disease of the CNS. In MS, inflammatory attacks against myelin, proposed to be autoimmune, cause myelin decay and oligodendrocyte loss, leaving neurons vulnerable. Current therapies target the prominent neuroinflammation but are mostly ineffective in protecting from neurodegeneration and the progressive neurological disability. People with MS have substantially higher levels of extracellular glutamate, the main excitatory neurotransmitter. This impairs cellular homeostasis to cause excitotoxic stress. Large conductance Ca2 ^+^-activated K ^+ ^channels (BK channels) could preserve myelin or allow its recovery by protecting cells from the resulting excessive excitability. This review evaluates the role of excitotoxic stress, myelination and BK channels in MS pathology, and explores the hypothesis that BK channel activation could be a therapeutic strategy to protect oligodendrocytes from excitotoxic stress in MS. This could reduce progression of neurological disability if used in parallel to immunomodulatory therapies.

MS is the most prevalent chronic demyelinating disease which affects 2.8 million people worldwide ^1, 2^. Its increasing prevalence poses a significant socio-economic burden. The aetiology of the disease is not completely understood, but demyelination pathology predominates alongside inflammation. In demyelinating diseases, like MS, an initial local attack against myelin sheath is proposed to trigger a cascade of neuroinflammatory and degenerative pathways causing damage to oligodendrocytes, myelin, and neurons ^3^. This impairs CNS conduction. Other less common demyelinating conditions such as Neuromyelitis optica (Devic's disease), transverse myelitis, and acute disseminated encephalomyelitis all have an inflammatory component ^4^. Demyelinating diseases may also have a genetic cause such as adrenoleukodystrophy, which is a X-linked genetic disorder where mutation in the *ABCD1* gene causes a defect in the corresponding ABCD1 transporter protein and accumulation of very long chain fatty acids in the brain and spinal cord leading to inflammation in the white matter, cerebral demyelination and neurodegeneration ^5^. Fragile X syndrome is a genetic disorder where transcriptional silencing of the *FMR1* gene leads to loss of the corresponding fragile X mental retardation protein (FMRP). FMRP acts as a RNA transcriptional regulator affecting the function of hundreds of proteins ^6^. Demyelination is an under-recognised feature of fragile X syndrome ^7^, in model systems FMRP silencing has been found to decrease the degree of myelination ^8^.

MS treatments can be classed as disease modifying therapies, (DMTs), to delay progression, or symptom management treatments. Current DMTs are immunomodulatory, with some specifically preventing myelin attack, notably by blocking peripheral immunity ^9^. Despite positive outcomes for neuroinflammation, underlying pathology is still not completely targeted ( Table 1). B cell directed therapies are amongst the most effective treatments; as these reflect the emerging disease understanding placing memory B cells at the centre of the disease mechanism ^10^. Although disability is reduced, neurodegeneration and defects in remyelination and repair still occur. Progression to secondary progressive MS (SPMS) is often not prevented and therapies successful for relapsing remitting MS (RRMS) become ineffective ^11^). With the difficulty of finding strategies to prevent neurodegeneration in general and few DMTs for SPMS, new therapeutic approaches need to target underlying demyelination, to date no remyelination strategies have proved effective ^12^. This unmet clinical need has led to the development of some diverse approaches using both repurposed drugs and novel therapeutics. Some of the most promising ideas are listed in Table 1. It is important to consider other targets that may protect mature oligodendrocytes from demyelinating pathologies like multiple sclerosis. One promising target is the the unfolded protein response (UPR). The UPR can work to preserve cell viability during periods of endoplasmic reticulum stress, like those caused by excessive inflammation, by temporarily halting translation to allow the re- establishment of protein homeostasis. Pancreatic endoplasmic reticulum kinase (PERK) is implicated in this. Increasing PERK activation in mouse models of MS protected myelin, in the presence of MS- related cytokine interferon- γ ^18^. This even improved survival of remyelinating oligodendrocytes and recovery in demyelinated lesions ^18^. A later study indicated that the PERK protective pathway can be enhanced by nuclear factor κB (NFκB) activation ^19^. Oligodendrocytes were rescued only in mice with MOG- induced EAE and PERK gene deficiency, whilst healthy oligodendrocytes remained unaffected ^19^. This might indicate this UPR signalling pathway specifically protects from inflammatory and demyelinating pathology. Studies have replicated these cytoprotective effects, but also indicated the need for targeted activation of NFkB ^19– 21^. Excessive generalised activation may worsen inflammation by dysregulating immunity and promoting autoimmune reactions, possibly leading to adverse effects ^22^.

**Table 1.** Summary of agents showing some promise in preserving myelin or as remyelinators.

Drug:	Type:	Mechanism of Action:	Reference:
Clemastine	First generation anti-histamine.	Potent activity against a wide range of GPCRs including histamine, muscarinic and adrenergic receptors. In the reBUILD trial the drug reduced visual evoked potentials latency (VEPs). Sedating and increased fatigue in the trial.	13
Metformin	Chemotherapeutic agent, approved for diabetes.	Metformin was found to reverse age-related changes, including repression of the UPR enabling oligodendrocytes to respond to differentiation factors. Currently in clinical trials for MS. (NCT04121468)	14
Bexarotene	Approved anti-cancer agent.	Retinoid X receptor agonist. Promotes OPC differentiation and remyelination. A phase 2a trial failed on the primary outcome measure but a statistically significant effect on the magnetization transfer ratio in submedial lesions. A poor side effect profile means the drug will not be pursued further.	15
Theophylline	Non-selective phosphodiesterase inhibitor. Approved drug for respiratory conditions.	The acetylated form protein Ac-eEF1A1 interacts with and removes the myelination/ remyelination transcription factor Sox10 from the nucleus. Theophyline activates the deacetylase HDAC2 to deacetylate eEF1A1 and restore myelination capacity. Progress to clinic is not yet reported.	16
Bazedoxifene	Third generation selective estrogen receptor modulator (SERM).	Enhances differentiation and remyelination of OPCs. Acts independently of its normal estrogen target. The enzyme 3β-hydroxysteroid-Δ8, Δ7-isomerase was identified as the potential target.	17

Alternatively, the leukaemia inhibitory factor (LIF), of the IL-6 cytokine family, was also proposed to protect mature oligodendrocytes from demyelination. Its levels determine growth and differentiation of inflammatory cells, but also possibly neural cells. Exogenous LIF protected against demyelination in cuprizone induced mouse models of MS with knockout endogenous LIF ^23^. Since cuprizone is toxic to oligodendrocytes but does not elicit an inflammatory response as seen in the EAE model, the authors indicated this method is directly cytoprotective ^23^. However, some studies suggest its activation might lead to negative effects on neuroinflammation, and state limited ability to cross the BBB when administered therapeutically; although its delivery and effectiveness significantly improved when using lentiviral vectors for CNS- specific expression in murine models ^23, 24^.

Similarly, agents aimed at attenuating the inflammation were also discovered to have independent neuroprotective effects which can lead to reduced demyelination. For example repurposing the antibiotic minocycline, which is thought to enhance anti-apoptotic signalling and was combined with atorvastatin to achieve an enhanced reduction in EAE severity ^25^. In a randomised controlled trial, taking minocycline within the first 6 months of the clinically isolated syndrome significantly decreased the risk of developing MS but not after two years, which may suggest a delaying instead of disease- modifying effect ^26^. The diabetic treatment metformin is known to repress the UPR via a miR-132 and PERK-eif2α dependent mechanism ^27^. The selective estrogen modulator (SERM) bazedoxifene has been shown to reduce ER stress and prevent activation of the UPR in β cells ^28^. In experimental MS, in the cuprizone model, bazedoxifene promotes remyelination apparently through activation of the integrated response pathway. While the mechanism of this agent’s action remains unclear it is in trials for MS ( Table 1).

Finally, in the MS lesion microenvironment, the excessive inflammation and failure of energy metabolism may lead to acidosis and subsequent activation of acid sensing ion channels (ASICs), which were found upregulated in both axons and oligodendrocytes in active MS lesions ^29^. These proton gated cation channels are activated in similar conditions of excitotoxicity as the BK channel. Amiloride or psalmotoxin-1, blocking respectively ASICs or ASIC1, protected oligodendrocytes from injury related to this acidosis, and the EAE mouse model with knockout ASIC1 had increased levels of myelin immunostaining compared to wild type ^29^.

The aims of this review are: to explain the importance of structurally and functionally intact myelin; to address the current lack of therapies targeting neurodegeneration particularly in MS; to evaluate the role of excitotoxicity in oligodendrocyte pathology and to explore the potential for therapeutic use of large conductance Ca ^2+^ activated K ^+^ channel activators to protect oligodendrocytes from excitotoxic stress, ultimately to preserve myelination.

## Oligodendrocytes and myelin in demyelinating disease

Neuronal impulse conduction is formed by action potentials (APs). These are generated from a momentary change in the ionic gradient across the axon membrane that propagates down and is relayed to the next neuron ^30^. Repeated and synchronised through billions of neurons, these rapidly transmit information across the body. In the CNS oligodendrocytes wrap axons with compact lamellae of their membrane myelin sheath ^31^. The low capacitance, high lipid content of myelin propagates action potentials (APs) directly onto short unmyelinated 1-μm axolemma segments, nodes of Ranvier. Voltage-gated Na ^+^ channels concentrate here to integrate a voltage difference so that APs can “skip” myelin internodes through saltatory conduction to increase velocity of impulses. The diameter of myelinated axons positively correlates with conduction velocity ^32, 33^; while myelin thickness inversely correlates with capacitance ^34^. Therefore, myelin provides an energy saving evolutionary adaptation; also because it restricts the number of Na ^+^/K ^+^ ATPases to the nodes, so it decreases the chemical energy ATP required to maintain resting potential ^31^. By myelinating larger axons, above ~2 μm in diameter, myelin allows signals to be transmitted fast over a long range ^31^.

**Figure 1.**
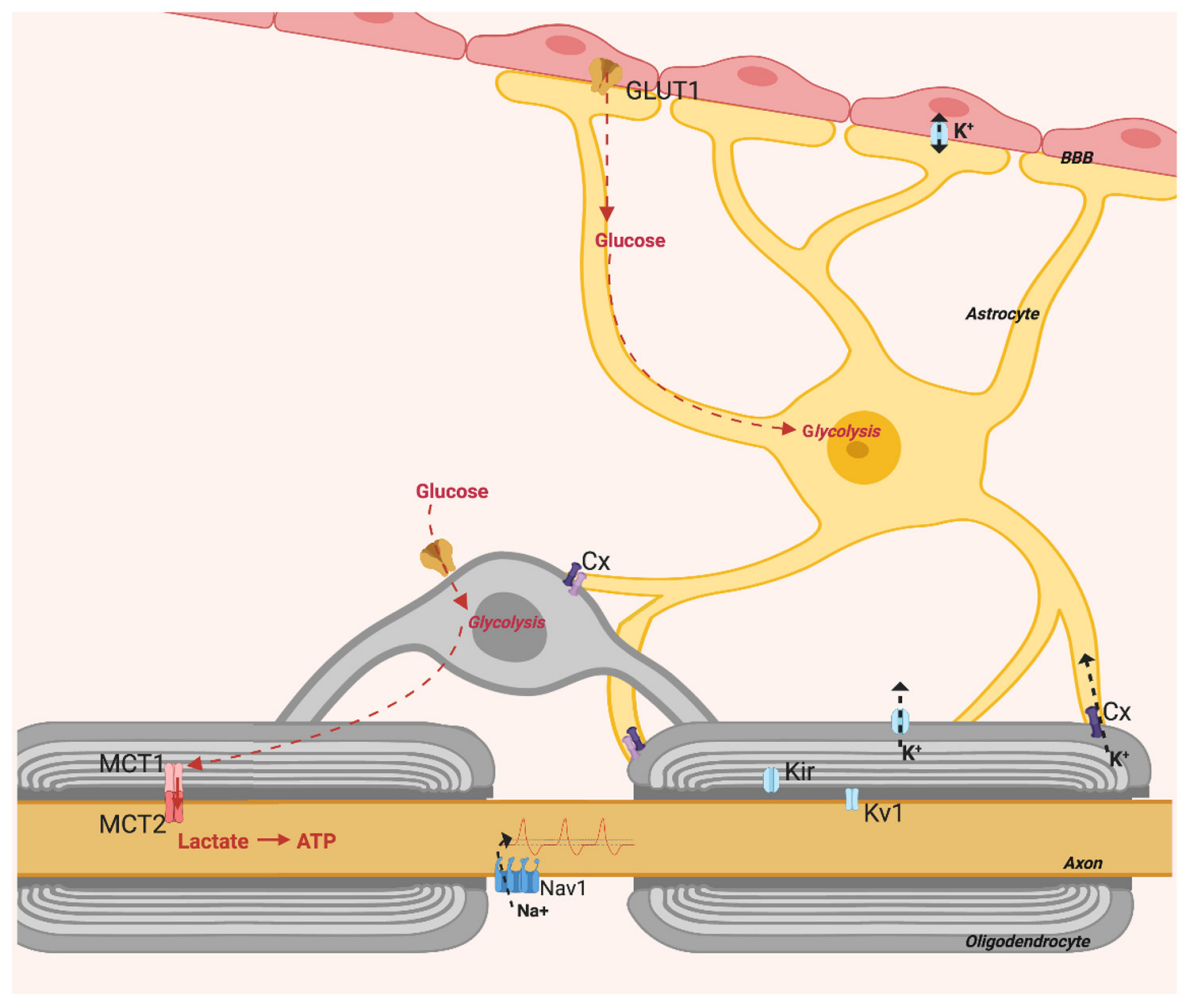
Oligodendrocytes provide axons with metabolic support, this is activity dependent and requires communication with astrocytes. Figure created with *BioRender*.

The brain expends one-fifth of total body energy output, but myelin prevents axons from receiving metabolic support extracellularly, so healthy oligodendrocytes are indispensable for axonal support ( Figure 1). Although neurons rely on their own mitochondria to synthesise ATP, these require glial glycolytic products, primarily lactate ^35^. Neuronal death can be induced by inhibiting oligodendrocyte glycolysis or neuronal mitochondrial respiration, but not by inhibiting neuronal glycolysis or oligodendrocyte oxidative phosphorylation ^36^. It was found that deleting the lactate transporter protein MCT1 impaired axons and caused atrophy ^37^. MCT1 being expressed relatively specifically by oligodendrocytes, these results indicate oligodendrocytes are important for healthy neuronal metabolism. However, other studies found that upon electrical stimulation neurons used their own glucose to synthesise energy, which might indicate oligodendrocytes are a primary glycolytic source only for neurons at rest ^38^. MCT1 is lost in neurodegenerative diseases like amyotrophic lateral sclerosis, where motor neuron death at the spinal cord indicates impaired axonal lactate supply ^37^. Neurons may depend on oligodendrocytes for metabolic support to survive and function properly, but the pathological relationship may cause damage before or separate to evident demyelination.

Metabolic support to axons requires astrocytes, which transfer glycolytic products to neurons through oligodendrocytes ( Figure 1). This by way of connexins on astrocytic processes and on oligodendrocytes which co-localise to form gap junctions ^39^. Astrocytes have glycogen stores and upon hypoxia or hypoglycaemia glycogen is catabolised into lactate for delivery to neurons ^40^. Furthermore, connexins found at paranodes may indicate a cooperation of astrocytes and oligodendrocytes in regulating axon electrical properties ^39, 41^ ( Figure 1). Astrocytes not only regulate axon activity and deliver metabolites; they also regulate BBB passage with astrocytic end feet that adjust membrane permeability. Healthy myelin preserves axon structure, metabolism and function, and potentially improves the general glia–axon relationship.

### Demyelination in multiple sclerosis

Demyelination is the erosion of myelin sheaths, which exposes nerve fibres leading to failure of impulse conduction. It can derive directly from traumatic or ischaemic injury ^42^. Alternatively it originates from attack of myelin related proteins in autoimmune disease ^43^. Loss of myelin does not necessarily lead to neuronal death, but overburdens axons by decreasing efficiency of energy homeostasis, making it harder for neurons to meet metabolic demands. Without myelin for saltatory conduction, energy needed to relay impulses increases. This eventually leads to increased functional impairment and susceptibility to further neurodegeneration.

**Figure 2.**
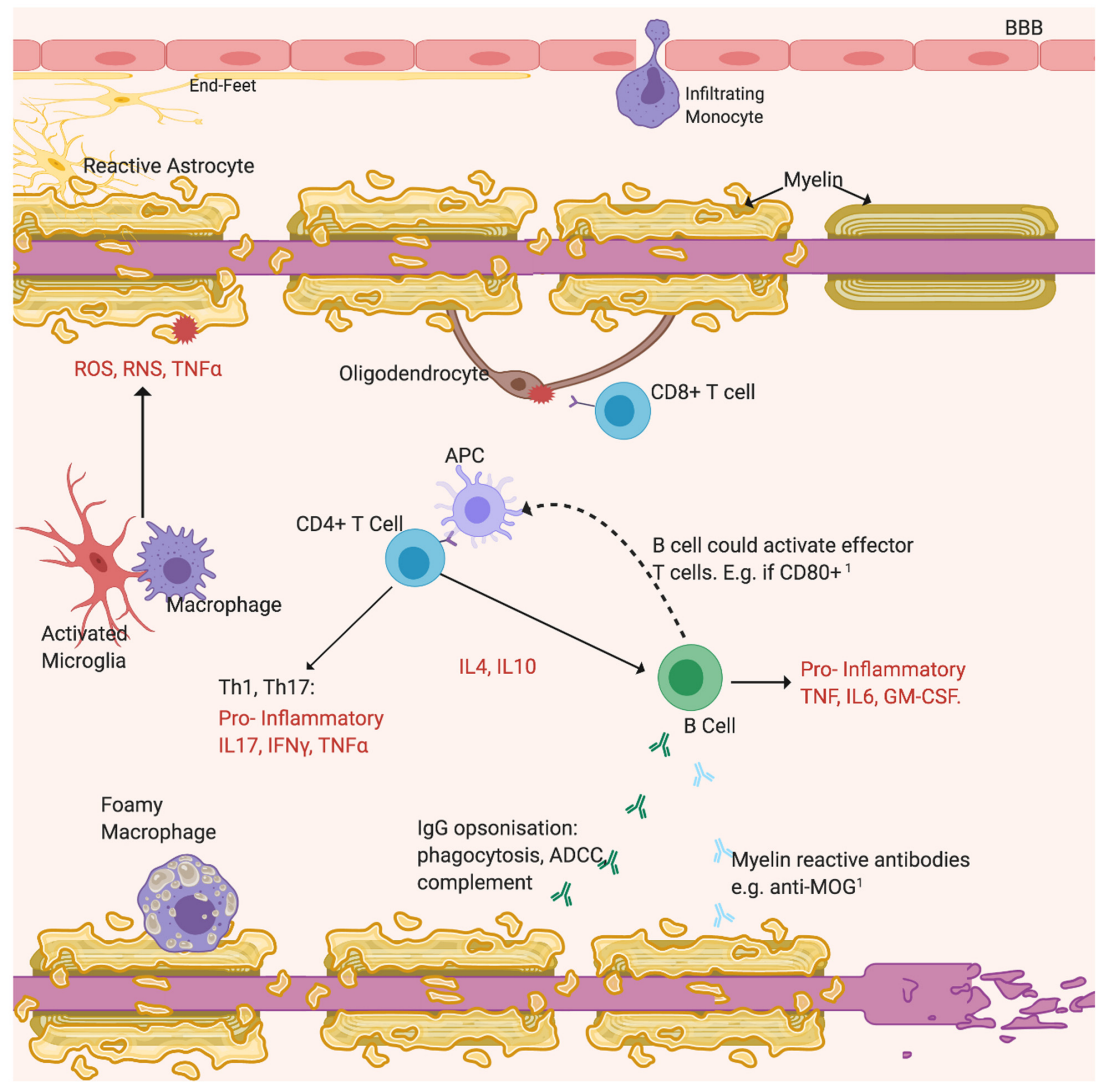
Demyelination may derive from antibody attack. Opsonisation by non-specific IgG activates the cytotoxic complement system and ADCC. The emerging importance of B cells is highlighted by recent findings ^44^. Additional roles include possibly secreting anti-myelin antibodies and acting as APCs to increase T cell activation 45, labelled 1. Cytotoxic CD8 ^+^ T cells react against self-antigens expressed by oligodendrocytes. Resident microglia or peripheral macrophages phagocytose myelin residues and debris. Reactive astrocytes, activated microglia and Th cells activated by APCs drive inflammation by secreting pro-inflammatory cytokines (TNFα, IFNγ, interleukins) and neurotoxicity by releasing free radicals (ROS, RNS) ^43^. Subsequently to myelin loss, axons degenerate. Figure created with *BioRender*.

The “sclerosis” of MS is the fibrotic lesion that forms in the brain or spinal cord from gliosis of astrocytes and microglia, often located near vasculature. The BBB appears “leaky” as shown by gadolinium-enhanced magnetic resonance imaging (MRI) from infiltration of blood-borne macrophages, T lymphocytes and B cells, which contribute to demyelination ^43^ ( Figure 2). After two temporally and spatially distinct acute inflammatory episodes, MS can diagnosed and is classified as relapsing–remitting or primary progressive MS depending on the disease course ^4^. As lesions become chronic, factors determining whether inflammation resolves and remyelination occurs are not fully understood. However, demyelination may share pathways with ischaemia and viral infection ^4^. Persisting inflammation and remyelination failure and nerve loss contribute to progressive MS ^11^. Without tissue repair, permanent loss of function often ensues.

Episodes may resolve incompletely and RRMS invariably involves neurological decline. Motor symptoms generally affect all patients eventually during disease course, but can involve sensory system particularly sight, pyramidal tracts, psychological aspects, brainstem and autonomic functions ^46^. Spinal cord lesions typically cause most of the lower limb disability and are both the white and grey matter ^47^, which contribute to the atrophy observed. This is observed early in MS brain and spinal cord when measured by atrophy using MRI, as an indicator of neurodegeneration ^48^. Associated neuroaxonal damage, measured as serum and notably intrathecal neurofilament, correlates with disability severity ^49^. Most patients eventually proceed to SPMS, notably those with significant early disease activity ^4^. SPMS develops when compensation pathways becomes exhausted and is notably associated with neurodegenerative state with progressive atrophy, enlarging lesions, chronic inflammation and remyelination failure.

### Preserving myelin 

Oligodendrocytes are limited in their ability to respond to damage and at least in part depend on replacement by their precursors, OPCs ^50^. In the adult CNS, NG2 ^+^ cells, which include OPCs and neural progenitors, constitute nearly 9% of white matter. Their migration into sites of injury is crucial for remyelination, whereby myelin regenerates spontaneously around demyelinated axons ^51^. Preserving myelin is important because neuroaxonal regeneration is limited. Macrophages have a strong influence, and microglia promote this by clearing myelin debris ^52^. Underlying demyelination and inflammation must resolve before new myelin forms. Remyelination may protect axons from inflammation-mediated neurotoxicity ^53^ and is observed in both acute and chronic lesions, even concomitant to demyelination, and in early MS ^43^.

Successful remyelination depends on sufficient OPC pools, their migration and survival, until differentiated into myelinating oligodendrocytes; but this does not guarantee it. In MS, OPCs differentiation may arrest before myelin synthesis completes ^42^. Axonal density is higher in remyelinated than chronic demyelinated plaques. However, demyelination may re-occur more frequently in new myelin because newly differentiated oligodendrocytes may produce thinner and shorter sheaths, possibly from external ischaemic factors of the neuroinflammatory environment impairing proper myelination ^54^. Additionally, lesion remyelination occurs 20% more often in acute than chronic lesions, so remyelination may inversely correlate with disease progression or age ^55^. Therefore, preserving myelin might provide a better neuroprotective strategy than remyelination.

## Communication between axons and myelin

About one-third of myelin sheath constitutes proteins that determine myelin architecture. Myelin basic protein (MBP) is a final component added to sheaths, responsible for compaction. MBP localises and draws two adjacent membranes together at clusters, where it forms a dense fibrillary network ^56^. This prevents signal dissipation and makes neurons more energy efficient. Downregulation of MBP impairs sheath structure *in vivo* and knockout decreases axonal calibre ^57, 58^. Conversely, 2',3'-Cyclic nucleotide 3'-phosphodiesterase (CNP) regulates cytoplasm quantity within myelin by maintaining actin cytoskeleton. This creates channels and directly counteracts MBP compaction ^59^. Working antagonistically, CNP and MBP can adjust these channel systems, possibly to modulate the type and amount of substrate exchange with axons. In mice, knockout of *CNP1* and of the functionally similar myelin proteolipid protein caused loss of fibres, of axonal integrity and axonal swelling by impairing neuronal transport ^58, 60^. This indicates that intact microstructure is important to preserve local support by oligodendrocytes and that effective conduction depends on this. Indeed, these properties of myelin microstructure can vary to preserve diverse neural networks, to adjust input latencies in nuclei. A latency of about 2 ms remains constant for thalamocortical signals to reach the sensory cortex despite fibres of different lengths ^61^. Instead of gross insulation, myelin tunes its microstructure to local axon requirements.

Electrically-silenced axons myelinate inadequately ^62^. Maintaining high K ^+^ levels extracellularly reduced myelination by increasing depolarisation duration, so APs are a putative channel of communication with oligodendrocytes ^63^. Recent advances in electron microscopy and myelin preservation revealed structure of the developed myelin sheath around axons ^64^. It is now possible to infer a peri-axonal space, where APs may be relayed by neurotransmitter release. Glutamate is the main excitatory neurotransmitter of the nervous system. Typically, it is released at axon terminals to bind to ligand gated ionotropic receptors found post-synaptically on dendrites. Types of ionotropic receptors are glutamatergic NMDARs, AMPARs and kainate receptors. Upon neurotransmitter binding these open voltage-gated channels for selective cation influx, for AP relay ^65^. In mature oligodendrocytes, glutamate may be released at the axolemma to affect the inner tongue of myelin sheath.

Glutamate release from synaptic vesicles along axons can stimulate MBP production to promote the insulating properties of myelin ^66^. Glutamatergic synapses are a feature of developing OPCs, whereby differentiating oligodendrocytes may depend on glutamate signalling for myelination ^67^. Activity dependent myelination may promote the migration and differentiation of OPCs. Stimulating the premotor cortex resulted in increased OPC migration and myelin thickness only in the optogenetically stimulated mouse model. This was associated with improved motor skills ^68^. Glutamatergic signalling downregulation may alter myelin thickness because, in mice, reduction of visual stimuli associated with reduced conduction velocity ^69^. When tetanus toxin was used to inhibit glutamate release from the synaptosome, Ca ^2+^ influx into oligodendrocytes did not occur ^70^, supporting mediation by excitatory neurotransmitter release. Although most myelinic ionotropic receptors are removed with differentiation, their use in preserving correct myelination in mature oligodendrocytes might explain the few remaining ^70^. NMDARs at the myelin sheath also gauge glycolytic delivery in response to axon energy demand ^71^. Substrate exchange may be triggered by AMPAR/ NMDAR activation, which induced exosome delivery ^72^. Dysregulation of firing frequency would therefore reduce myelinic neuronal support ( Figure 1). In pathology this activity dependent alteration of myelin architecture may be rendered unresponsive, counterproductive or even toxic to neurons, even before overt demyelination. Modulating this glutamatergic signalling may preserve myelin and neurons.

## Excitotoxic stress

### Excessive extracellular glutamate

Excitotoxic stress is caused by excessive or prolonged activation of glutamatergic receptors causing Ca ^2+^ overload. This sustains pro-apoptotic pathways involving enzymes and transcription factors like MAPK and NF-κB, which degrade membranes, proteins and intracellular organelles. Increased glutamatergic signalling can be triggered by the energy deficiency from the cellular damage in lesions, mitochondrial dysfunction and oxidative stress ^73, 74^. The last involves highly reactive and damaging free radicals: ROS and RNS. These cause mitochondrial membrane damage by lipid peroxidation, which exacerbates cellular burden and glutamatergic signalling ^75^. At high levels glutamate is thought to induce oxidative stress by means of blockade of the glutamate/cystine antiporter (XC–Cys/Glu) that prevents uptake of cystine and synthesis of the anti-oxidant glutathione, in a form of cell death termed ferroptosis or oxytosis ^76^.

Damage to neurons causes axon swelling, where ion channels including voltage-gated sodium channels are upregulated to attempt compensation for impaired conduction ^77, 78^. Excitotoxic damage to myelin may cause this upregulation without necessarily causing overt demyelination ^78^. Axon swelling impairs network connectivity in MS, where sustained glutamatergic activation associates significantly with increased neurological disability ^79^.

Glutamate is upregulated in MS CSF (p<0.001) and carrying the polymorphism rs794185 that further increases this associates with neurodegeneration ^79, 80^. The major source of glutamate production is difficult to discern, but evidence suggests neuroinflammation is important. Pro-inflammatory cytokines TNFα and IL-1β cause neurotoxicity by downregulating astrocytic glutamate transporter and glutaminase which accumulates glutamate in the extracellular space ^73, 81, 82^. IL-1β but not TNFα are established as significantly upregulated in MS CSF ^82, 83^. Immune activation upregulated the cystine glutamate exchanger on macrophages and microglia and in MS patients ^84^. To synthesise important antioxidant glutathione this exchanger releases glutamate extracellularly.

Table 2 describes drugs targeting excitotoxicity in MS, highlighting the still unmet clinical need. These therapies are inadequate clinically because antagonists of glutamatergic pathways can downregulate excitatory CNS conduction, which importantly can cause serious adverse events. Selectivity could be improved by targeting receptor subunits specific to glial cells and that are more permeable to pathological Ca ^2+^ accumulation, like NR1 and NR3 NMDAR subunits ^85^. Sodium channel blockers provide an alternative means to control excitotoxicity and some benefit has been noted in the more recent clinical trials, but they are poorly tolerated leading to non-compliance ^86, 87^.

**Table 2.** List of completed clinical trials to lower excitotoxicity and investigate neuroprotection in MS.

Drug:	Action:	Primary Outcome:	Results:	Study Reference:
Memantine	NMDAR antagonist.	Cognitive Impairment.	Stopped due to worsening neurological deficits.	90
Riluzole	Inhibits glutamate release from synaptic terminals; NMDA and kainate receptors modulator; keeps VGCCs inactivated.	Brain atrophy.	No significant difference compared to placebo (p= 0.065).	91
Memantine	NMDAR antagonist.	Spasticity.	No significant difference (p= 0.65).	92
Amantadine	NMDAR antagonist.	Fatigue.	Reduced compared with placebo (p< 0.05).	93
Amiloride, Riluzole, Fluoxetine	Respectively: reduce pro-apoptotic axonal Ca ^2+^ overload; glutamate mediated excitotoxicity; increases astrocytic lactate release to support neuronal energy metabolism.	Brain atrophy.	No significant difference (p= 0.99).	94
Lamotrigine	Sodium channel blocker	Cerebral volume loss.	At 24 months, no significant reduction in cerebral volume loss with lamotrigine compared with placebo.	86
Phenytoin	Sodium channel blocker	Thickness of retinal nerve fibre layer.	A 30% reduction in the extent of retinal nerve fibre layer loss with phenytoin at 6 months compared with placebo.	95
Oxcarbazepine	Sodium channel blocker	CSF NFL reduction.	Oxcarbazepine had no significant effect on CSF NFL levels, an effect on EDSS and MSWS scores was noted.	96

### Oligodendrocytes are deficient in their response to excitotoxic stress

Oxidative damage to proteins and lipids is substantially increased in acute demyelinating lesions compared to healthy white matter. Hypertrophic astrocytes and foamy macrophages are able to limit this damage by upregulating antioxidant superoxide dismutase, but not other components of lesion tissue including neurons and oligodendrocytes ^88^. Oligodendrocytes have a particularly inefficient antioxidant protection. These have a reduced ability to synthesise glutathione ^89^ and their death positively correlates with concentration of the highly reactive lipid peroxidation product 4-HNE ^97^. Oligodendrocytes are also the main cells that store iron in a balance that is susceptible to conversion to its oxidative divalent form ^98^. Their susceptibility to excess glutamate activation specifically is supported by *in vitro* studies. Only upon inhibition of glutamatergic receptors in oligodendrocytes-only cultures were the apoptotic indicators DNA fragmentation and caspase-3 abolished ^82, 99^.

Experimental autoimmune encephalomyelitis (EAE) is an established MS model induced by adoptive transfer of anti-myelin protein T cells. In EAE mice, 60% more of the oligodendrocytes population was preserved with the AMPA/kainate receptor inhibitor NBQX compared with administering phosphate buffered saline (PBS) only, which also improved neurologic impairment score (p <0.01) ^100^. AMPAR-mediated Ca ^2+^ influx activates a sustained phosphorylation of ERK1/2 to activate proapoptotic pathways in oligodendrocytes and mitochondrial impairment in a manner similar to ischaemia ^74^. Ca ^2+^-permeable AMPARs are upregulated only at MS lesions, but not in regions of healthy tissue ^101^, so Ca ^2+^ permeability might indicate upregulation of excitotoxic responses with demyelination. Considering the complex pathological microenvironment of lesions, glutamatergic receptor inhibition alone might not prevent cytotoxicity locally in MS. Pro-inflammatory damage spreads centrifugally from the lesion centre ^4^, so inhibition might instead prevent spread of excitotoxins.

AMPAR/kainate receptors are mainly expressed on oligodendrocytes soma, while myelin mainly expresses NMDARs ^102^. Excitotoxic stress to myelin can cause decompaction of myelin sheath ^103^, which can impair neuronal metabolism before overt demyelination. Since damaged or degraded myelin sheaths increase neuronal metabolic burden and expose axons to inflammation related toxins, this suggests therapeutically protecting myelin from excitotoxic stress may be neuroprotective in MS. A characteristic feature of MS is a dying back oligodendrogliopathy which, in a similar way to complement activation by direct antibody attack ^4^, might also be caused by activation of catalases and mitochondrial redox damage at myelin processes which retrogradely affects oligodendrocytes.

NMDARs induce weaker Ca ^2+^ currents compared with AMPARs but sustain these for longer ^65^. The small cytosolic compartment of myelin may quickly accumulate Ca ^2+^ concentrations sufficiently high to be toxic. All compartments needed for NMDARs to be functional have been detected with immunoblotting: NR1, NR2 and NR3 ^102^. These require activation by both glutamate and its co-agonist glycine. Release of only glutamate from myelinated axolemmas has been established ^70^. The Mg ^2+^ block characteristic of NMDARs can be released by a slight depolarisation ^65^, which may justify the expression of AMPARs on myelin at lower concentrations. Especially because AMPARs inhibitors only partially abolished the Ca ^2+^ current through myelin, but completely at oligodendrocytes soma, while non-selective ionotropic receptor inhibitor completely abolished at both locations ^102^. This suggests a mediating effect by AMPAR.

However, no significant decrease of NMDAR mediated Ca ^2+^ into oligodendrocytes when their inhibitors, NBQX or D-AP5 respectively, were added after ischaemia ^103^. The authors proposed excitotoxicity does not derive directly from glutamatergic Ca ^2+^ influx, but from the resulting K ^+^ and H ^+^ increase because the NMDA evoked current correlated with K ^+^ increase. The resulting decrease in pH (from K ^+^ and from the hypoxic cell) might activate H ^+^-gated TRP channels which then caused about 70% of the Ca ^2+^ rise ^103^. TRP block reduced myelin decompaction, so it is possible these channels are more responsible for the ischaemic excitotoxicity to oligodendrocytes than direct ionotropic receptor activation. Alternatively, the majority of Ca ^2+^ may derive from a secondary source, such as from subsequently activated voltage gated calcium channels (VGCCs) or the reversal of the Na ^+^/Ca ^2+^ exchanger which can occur in conditions of excessive depolarisation ^101^.

Dying oligodendrocytes release high levels of Fe ^2+^ which directly contributes to oxidative injury to neurons ^98^. This accumulates at acute demyelinating lesions, phagocytosed and released through oxidative burst. Ferrous iron, Fe ^2+^, is a mediator of the Fenton reaction that synthesises hydroxyl and H _2_O _2_ radicals ^98^. Excitotoxic stress will damage oligodendrocytes, which will in turn release more oxidative stress, although contribution of oligodendrocytes excitotoxicity is still unclear because complex to quantify.

## BK channels reduce excitotoxic stress

### BK channels

Large conductance calcium-activated, voltage gated potassium channels (BK channels) are the most diverse within the family of transmembrane protein channels, which also includes small and intermediate K ^+^ conductance (SK and IK) channels ^104^. These are activated by thresholds of voltage or Ca ^2+^ transients and accordingly control membrane potential by mediating efflux of the required amount of hyper-polarising K ^+^^105^. They can also be activated by other metal ions such as Mg ^2+^, but also by pH, arachidonic acid and nitric oxide. Encoded by the KCNMA1 (or SLO) gene, BK channels constitute a heterodimer of pore-forming α-subunits and a monomer comprising a voltage-sensing and a calcium-sensing module ^106^. Ubiquitous, BK channels are overexpressed in regions of high Ca ^2+^ concentrations ^107^. By mediating K ^+^ transients out of cells, BK channels can also regulate K ^+^ homeostasis, cell volume, and therefore have various functions including neuronal excitability, smooth muscle relaxation, blood pressure control and electrical tuning of cochlear hair cells ^108^.

The highly dynamic physiological properties of BK channels are partly due to the numerous α-subunit splice variants, which makes their translated protein structure highly versatile physiologically. For example, a cysteine-rich 59-amino-acid insert between RCK domains called STREX variant can be added to the C-terminus ^109^, resulting in increased sensitivity to activation, inducing higher neuronal firing frequencies. Additionally, BK channels assemble auxiliary subunits, such as β subunits (β1–4) ^110^. These can modify activity, including modifying sensitivity to its activators, voltage or Ca ^2+^, or by activating protein kinases ^111^. Furthermore, the association with γ subunits, which are leucine rich repeat containing proteins, can increase stimulability of the BK channel by decreasing the negative voltage difference threshold ^112^. Ultimately, this increases the range of pharmacological applications of these channels.

### BK channels regulate neuronal excitability

In the CNS, BK channels are abundantly expressed on axons, dendrites, soma and synaptic terminals in widespread CNS regions. Here, these can control the fast phase of after-hyperpolarisation. Additionally, these can control AP output by changing the magnitude and duration of incoming Ca ^2+^ spikes at dendrites ^113^. This will determine AP duration and firing frequency ^114^. BK channels can mediate their activities and their responses specifically for their cellular location and type of neuronal cell by co-localising with functionally distinct VGCCs ^114^. BK channels have been shown to co-localise with L-/, P/Q-, or N-/ types of VGCCs ^115, 116^. Depending on the frequency of basal firing, the BK channels at that neuronal cell will typically provide the opposite effect to modulate and re-set the phase, ultimately to flatten the frequency-current curve and control neuronal excitability. This would occur in a manner similar to hyperpolarisation activated by cyclic nucleotide gated channels, that set the “pacemaker” firing frequency in the brain ^117^. Overall, studies of BK channels indicate these tune the neuronal signal by amplifying it if weak or reducing it if too strong, rather than stringently enhance inhibition or excitation ^118– 120^. 

BK channels also have an important role in directly mediating neurotransmitter release, this is supported by their co-localisation to VGCCs with those of the P/Q-type being most frequently observed. This co-localisation occurs predominantly at dendrites where it regulates dendritic spike generation relative to neurotransmitter release ^121^. This is consistent with localisation of the BK α subunits at presynaptic terminals in functionally important axon tracts ^122^. At these locations, BK channels limited the Ca ^2+^ mediated neurotransmitter release by decreasing presynaptic APs duration ^122^. Indeed, release of neurotransmitter from vesicles is triggered by Ca ^2+^ elevated locally through VGCCs, once the propagated AP reaches the terminal ^123^. Typically, BK channels would reduce neurotransmitter release, because these are able to reduce the amplitude of the presynaptic AP. An important demonstration of this is the effect on neurotransmitter release by CA3 hippocampal neurons and associated APs upon addition of BK channel blockers. The resulting spontaneous EPSCs increased in amplitude and frequency ^122^. This inhibition ultimately reduces release of glutamate, but does not occur for inhibitory neurotransmitter GABA ^124^. Therefore, BK channels are key to avert overexcitation of the post synaptic neuron.

### Mediators of excitotoxic stress

Physiologically, BK channels can prevent too much neurotransmitter from causing excessive depolarisation and Ca ^2+^ accumulation post-synaptically. In mice where acute focal cerebral ischemia was induced by middle cerebral artery occlusion, the neurological symptoms were significantly higher with knockout of the BK α subunit compared to wild type ^125^. This may imply glutamate-induced oxidative stress, and consequences for acute and chronic neurodegeneration. This negative feedback by BK channels might only occur if propagated APs are high enough to induce levels of intracellular Ca ^2+^ and neurotransmitter similar to those observed in pathological conditions. For example, only upon addition of 4-AP, a non-specific inhibitor of voltage gated K ^+^ channels, were BK channels activated to decrease AP amplitude post-synaptically and decrease neurotransmitter release ^126^. No amplified repolarisation or reduced neurotransmitter release by BK channels was observed without 4-AP. This is specific to excitatory neurotransmitter release, because a concentration dependent reduction in ischaemia mediated by NMDAR correlated with increased opening of BK channels by the activator NS1619 ^127^. By creating a negative feedback control to disproportionate neurotransmitter release, BK channels may be an emergency break to prevent hyperexcitability and subsequent toxicity.

**Figure 3.**
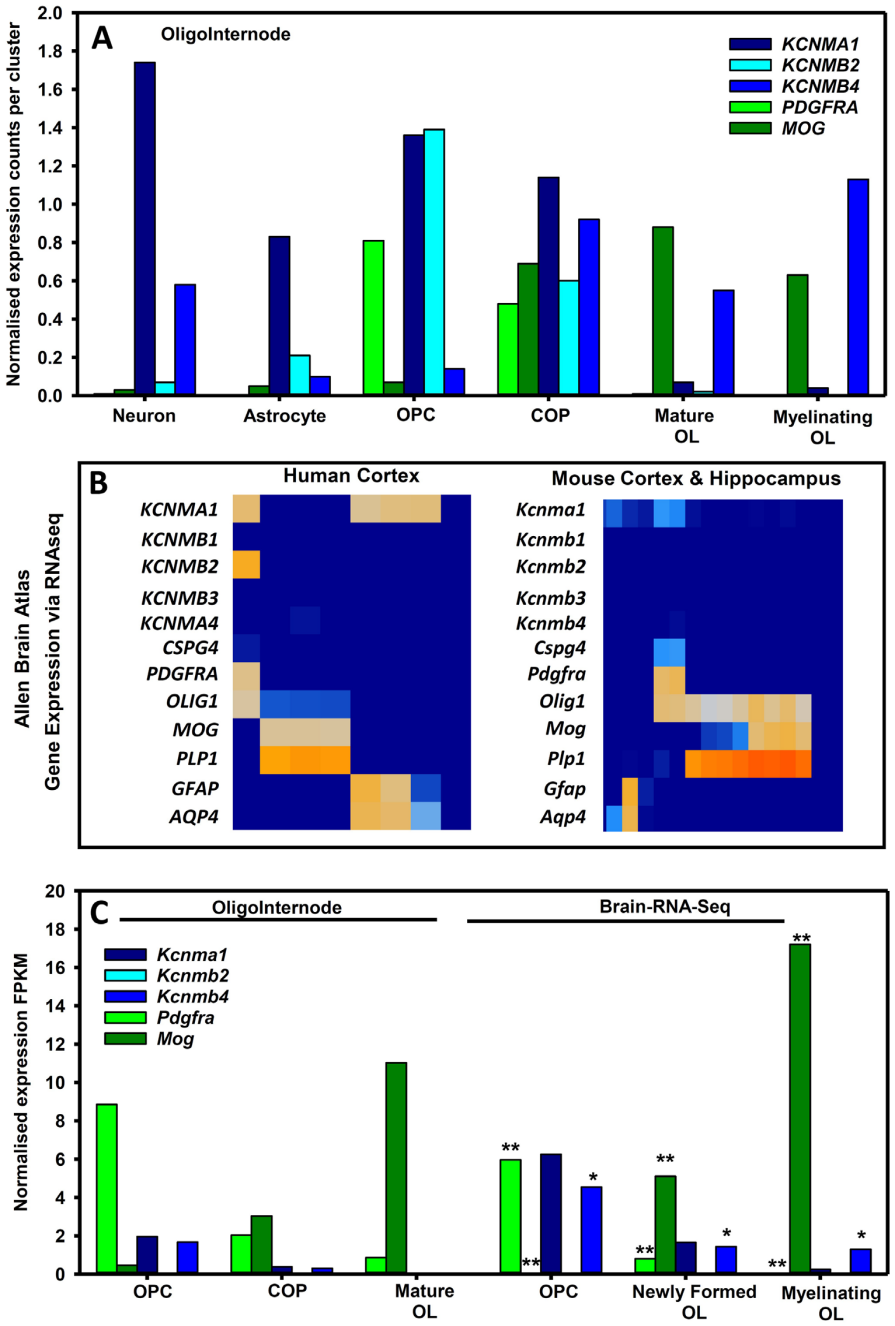
Oligodendrocyte expression of BKCa channels in humans and mice. The expression of: BK channels; platelet-derived growth factor receptor alpha (PDGFRA) and chondroitin sulphate proteoglycan four (CSPG4/NG2) as markers for oligodendrocyte precursor cells (OPC) and committed oligodendrocyte precursors ((COP); myelin oligodendrocyte glycoprotein (MOG) and proteolipid protein one (PLP1) as markers of mature oliogdendrocytes (OL); human glutamatergic neurons (Neuro2 GAD2 0.02, SLC17A7 2.11 (Jäkel *et al.* 2019); and aquaporin 4 (AQP4) and glial fibrillary acidic protein (GFAP) as markers for astrocytes channels was extracted from public data bases ( **A**) Expression of BK channels in human cells in human white matter tissues extracted from the oligointernode ( https://ki.se/en/mbb/oligointernode ^135^. ( **B**) Expression of human and mouse BK channels from cortical brain tissue using 10X single cell RNAseq from the Allen Brain Atlas ( www.portal.brain-map.org) ( **C**) BK expression in OPC and mature oligodendrocytes from RNAseq data from the Oligointernode portal ^136^ and the Brain RNA-Seq portal ( www.brainrnaseq.org ^137^). Data is expressed as fragments per kilobase of transcript per million mapped reads (FPKM). * = data values reduced 10 times ** = data values reduced 100 times.

Based on the reviewed evidence, there is an association of BK channel subunits with disease. BK channel activation is neuroprotective in animal models of spinal cord injury, ischaemia and excitotoxic stress ^45, 125, 127, 128^, which share some pathological similarities with MS. This is confirmed by allele knockout or blockage with the antagonists iberiotoxin and/or paxilline that exacerbated injury ^125, 127^. The agonist isopimaric acid targeted the α subunit to improve motor function in rats with spinal cord injury ^45^. Similarly, NS1619 activated subunit α1 to effectively increase neuroprotection in rat and mouse cortex when used with leptin ^127^. Certain subunits may only be activated and therefore effectively targeted in the presence of cellular injury or excitotoxic conditions ^126, 128^, a quality which could arguably improve specificity of an agent to injured tissue. However, these studies focused on neuron expression of BK channels and axon rather than oligodendrocyte integrity as outcome. In clinical trials, BMS-204352 activates KCNMA1, but also activates another potassium channel KCNQ. This was trialled as a safer neuroprotective agent to reduce intracellular Ca ^2+^ levels in acute ischaemic stroke, but did not significantly improve outcome in this study ^129^. Andolast has been shown to significantly improve asthma symptoms compared to placebo, but its subunit targets have not yet been investigated ^130^. The anti- glaucoma agent unoprostone isopropyl activated iberiotoxin resistant BK channels, therefore likely successfully targeted the β2, or β3, subunits ^131^. The β regulatory subunit is generally quite tissue specific and therefore arguably a better target for an agonist than the α- subunit ^132^. In the CNS, KCNMB4 and KCNMB2 are the main regulatory subunits expressed ^133^. VSN16R, which is thought to target preferentially the β4 subunit, has shown promise in reducing spasticity at higher doses with few side effects ^133, 134^. Notably, KCNMB4 is expressed in human mature oligodendrocytes ( Figure 3) and is possibly more abundant in chronic inactive MS lesions 135. Evidence is needed to ascertain whether an agonist to KCNMB4, or to another regulatory subunit, changes outcome in MS pathogenesis by targeting oligodendrocytes and by preserving myelin. Recently Fragile X syndrome the major genetic cause of intellectual disability has been shown to have a demyelinating component. BK channel activation (specifically α1β4) has been shown to restore both electrophysiological and behavioural functions in experimental Fragile X.

### Activating BK channels to protect oligodendrocytes

Much of the available evidence relates to neurons, but if there is a functional link between the role of BK channels and oligodendrocytes in mediating this excitotoxic stress, targeting this could possibly provide an avenue for disease modifying therapy in MS.

Although BK channels, notably KCNMB4 isoforms are neuronally expressed ^138^, it is evident that KCNMB4 is also present and differentially expressed by oligodendrocytes ^135, 139^. OPCs were associated with high expression of *KCNMA1* and *KCNMB2* ( Figure 3A. 3B), at a time when they express many ion channels perhaps as part of the pre-myelination glial-neuronal synapse ^140^. However, it is evident that oligodendrocyte maturation and myelination was associated with their relative loss and the upregulation of the KCNMB4 BK isoform ( Figure 3A, 3B). In addition transcriptomic expression of KCNMA1 and KCNMB4 in NG2+ cells has been found ^141^.

Human KCNMB4 expression increases as OPCs mature into oligodendrocytes and was increased in myelinating oligodendrocytes ( Figure 3A). This is perhaps consistent with elevated KCNMB4 expression in chronic inactive multiple sclerosis lesions ^135^. In contrast mouse OPC and oligodendrocytes do not seem to express much *Kcnmb2* ( Figure 3B, 3C). However, as occurs in humans, *Kcnma1* is most marked in the OPC and is down-regulated as oligodendrocytes mature and myelinate ( Figure 3C). Likewise, *Kcnmb4* can sometimes be found at higher levels in OPCs, but persists in mature oligodendrocytes to be the dominant BK channel isoform ( Figure 3B, 3C). *Kcnmb4* is expressed on the cell membrane and is also expressed in mitochondria ^132^. Loss of *Kcnma1* message during development is consistent with protein expression and functional calcium-induced signalling activity ^139^ and may play a role in oligodendrocyte differentiation.

Additionally, electrophysiological recordings of increased oligodendrocytes depolarisation corresponded to the increased intracellular fluorescence from labelled Ca ^2+^ upon glutamate-induced stimulation; which occurred only when the BK channel blocker iberiotoxin was added ^139^. This suggests a role of BK channels to regulate Ca ^2+^ influx to protect oligodendrocytes from excitotoxic stress. Other evidence indirectly supports this. As such the fundamental subunits of the NMDARs, NR1, NR2 and NR3 co-localise with myelin protein from primary optic nerve oligodendrocytes upon immunohistochemical staining ^102^. Blocking NMDARs substantially blocked myelin damage upon chemically induced ischaemia *in vitro* ^102^. This was the first evidence of axo-myelinic signalling, indicating that glutamate released from the axon can cause Ca ^2+^ to enter oligodendrocytes through the myelin sheath. Importantly, it has been found that mature oligodendrocytes express NMDARs, and that small quantities of excitatory neurotransmitters diffusing between axon and myelin could form sufficiently high concentrations to give rise to large Ca ^2+^ transients within mature oligodendrocytes ^142^. In health, oligodendrocytes already communicate with axons through NMDAR for trophic support ^71^ and BK channels form complexes with this receptor ^143^. Therefore, when activated, BK channels could protect oligodendrocytes from axon-induced excitotoxicity by increasing hyperpolarisation. Prolonging APs may increase the duration of the desensitised state of ionotropic channels and VGCCs to limit Ca ^2+^ influx. In demyelinating pathology, the excessive excitotoxicity could inhibit the endogenous protection by BK channels to oligodendrocytes. The addition of an activator could re-open these, re-establishing protective effects. A counter argument is that high extracellular potassium is primarily responsible by increasing length of neuronal depolarised state. Damaged oligodendrocytes may have a dysfunctional inward rectifier potassium channel, so K ^+^ clearance is faulty ^144^. Large levels of excitatory stimulation of myelin may result because when neurons are demyelinated or damaged they upregulate sodium channels, and subunits which maintain the depolarised state ^77^. In this scenario, BK channel activators might be counter-productive by increasing extracellular K ^+^, but possibly only if K ^+^ clearance is faulty.

BK channel activators could be used therapeutically to preserve function in demyelinating diseases, particularly MS. As described above, currently the standard treatment for MS targets inflammation, but curbing the pathological attack by the immune system does not protect from demyelination or excitotoxicity. Therefore, it does not prevent neurodegeneration or restore functionality lost ^11^. In MS, BK channels are expressed in both myelin and the axons it covers. Crucially, in chronically injured white matter, their activation upon Ca ^2+^ influx was observed only upon axon exposure subsequent to chronic spinal cord injury ^128^. Addition of the BK channel activator isopimaric acid preserved myelination after spinal cord injury in rats ^45^, where functionality correlated with preserved myelinated tracts. This suggests that a BK channel activator could target demyelination to preserve functionality in MS.

Only a few BK channel activators have been studied in the clinic, BMS-204352 (Maxipost) was developed for stroke while andolast is reported to be in phase III for asthma ^129, 130^. Unoprostone isopropyl is an atypical prostanoid used topically in the treatment of glaucoma ^131^. VSN16R was recently trialled in people with MS for muscle spasticity ^133, 134, 138^. This trial focussed on spasticity endpoints up to a week after administration of the drug and no remyelination parameters were studied ^134^. 

## Conclusion

There are numerous ways excessive glutamate may cause oligodendrocytes toxicity in demyelinating pathology. Neuroinflammation increases neuronal signalling which will damage neurons, that will release even more glutamate. The vicious cycle of damage by oxidative stress to cellular metabolism will exacerbate pathology. Close proximity to neurons, glutamatergic receptor expression and high vulnerability to oxidative stress makes oligodendrocytes particularly susceptible to excitotoxicity compared to other lesion tissue ^73, 74^. Oligodendrocytes perivascular location, as part of white matter, further increases this susceptibility, especially in MS where neuroinflammatory oxidative stress is central to demyelination.

BK channels can modulate cellular excitability and are even proposed to protect cells from release of excessive levels of excitatory neurotransmitters, by pairing with ionotropic glutamate receptors and VGCCs. It is plausible that BK channels could protect oligodendrocytes from excitotoxicity, supported by their expression in these cells ^139^. With high levels of glutamate BK channels become inactivated, possibly explaining their inability to protect cells in models of demyelination ^45^. It is therefore feasible that BK channel activators might protect pathological oligodendrocytes from excitotoxic stress. Considering oligodendrocytes primary function is axon myelination, then if BK channels preserve oligodendrocytes integrity myelination would also be preserved.

There is still little evidence of the functions of BK channels on oligodendrocytes and the involvement of BK channels in MS is an angle of research that has yet to be explored extensively. Therefore, *in vitro* tests are fundamental to establish a first functional link between BK channels, oligodendrocytes, oxidative stress and myelin preservation to verify the importance of conducting these investigations and possibly prompt more. Crucially, it is important to determine whether BK channels are expressed by oligodendrocytes, whether this expression depends on developmental stage, but also effects of glutamate-induced excitotoxicity in the context of myelination and the ability to target BK channels *in vivo*. This would define whether increasing the open conformation of BK channels with activating agents is a promising neuroprotective therapy to be used in parallel to immunosuppressive agents for the treatment of MS. 

## Abbreviations

ADCC: antibody- dependent cellular cytotoxicity

AMPAR: alpha-amino-3-hydroxy-5-methyl-4-isoxazolepropionic acid receptor

AP: action potential

APC: antigen presenting cell

ATP: adenosine triphosphate

BBB: blood brain barrier

BK: big conductance Ca ^2+^ activated K ^+^ (channel)

CD: cluster of differentiation

CNP: 2',3'-cyclic nucleotide 3'-phosphodiesterase

CNS: central nervous system

CSF: cerebrospinal fluid

Cx: connexin

DMEM: modified minimal essential medium

DMT: disease modifying therapy

DNA: deoxyribonucleic acid

EAE: experimental autoimmune encephalomyelitis

ERK: extracellular-signal regulated kinase

FBS: foetal bovine serum

GABA: gamma-aminobutyric acid

GAPDH: glyceraldehyde 3-phosphate dehydrogenase

GFAP: glial fibrillary acidic protein

GLUT1: glucose transporter 1

GM-CSF: granulocyte- macrophage colony stimulating factor

IFNγ: interferon gamma

IgG: immunoglobulin G

IL: interleukin

KCNM: Ca ^2+^-activated-K ^+^ channel subunit

Kir: inward rectifying K ^+^ channel

KO: knockout

Kv1: voltage gated K ^+^ channel

MAG: myelin- associated glycoprotein

MAPK: mitogen- activated protein kinase

MBP: myelin basic protein

MCT: monocarboxylate transporter

MDA: malondialdehyde

MOG: myelin oligodendrocyte glycoprotein

MRI: magnetic resonance imaging

MS: multiple sclerosis

Nav1: voltage gated Na+ channel

NFκB: nuclear factor kappa-light-chain-enhancer of activated B cells

NMDA(R): N-methyl-D-aspartate (receptor)

OPC: oligodendrocyte progenitor cell

PBS: phosphate-buffered saline

qPCR: quantitative polymerase chain reaction

RNS: reactive nitrogen species

ROS: reactive oxygen species

RRMS: relapsing- remitting multiple sclerosis

SPMS: secondary progressive multiple sclerosis

STREX: stress-axis regulated exon

TBARs: thiobarbituric acid reactive substance assay

Th: T helper (cell)

TNFα: tumour necrosis factor alpha

TNFβ: tumour necrosis factor beta

TRP: transient receptor potential (channel)

VGCC: voltage gated Ca ^2+^ channel

4-AP: 4-aminopyrimidine

## Data availability

### Underlying data

No data are associated with this article.
