# Supplementary material for: Oligodendrocytes, BK channels and the preservation of myelin
Source: F1000Res. 2021 Nov 15;10:781. Originally published 2021 Aug 9. [Version 2] doi: 10.12688/f1000research.53422.2 (PMC8596180; doi:10.12688/f1000research.53422.2)
Supplement: Peer review history

# Peer review: Reviewer response for version 2

**DOI:** 10.5256/f1000research.78993.r100202

**Published:** 2021-11-16

**Authors:** Wensheng Lin

**Affiliations:** 1 Department of Neuroscience, University of Minnesota, Minneapolis, MN, USA

No further comments.

Is the review written in accessible language?

Yes

Are all factual statements correct and adequately supported by citations?

Yes

Are the conclusions drawn appropriate in the context of the current research literature?

Yes

Is the topic of the review discussed comprehensively in the context of the current literature?

Yes

Reviewer Expertise:

oligodendrocyte biology and pathology

I confirm that I have read this submission and believe that I have an appropriate level of expertise to confirm that it is of an acceptable scientific standard.

# Peer review: Reviewer response for version 1

**DOI:** 10.5256/f1000research.56803.r94510

**Published:** 2021-09-27

**Authors:** Wensheng Lin

**Affiliations:** 1 Department of Neuroscience, University of Minnesota, Minneapolis, MN, USA

This review summarizes the current literature on the role of excitotoxic stress, myelination, and BK channels in MS pathology. The topic is timely and important. The manuscript is well written. However, there are a couple of concerns:

1. The authors stated that "preserving myelin might provide a better neuroprotective strategy than remyelination". In fact, this manuscript concentrates on demyelination, rather than remyelination. It could be better to replace remyelination with demyelination in the title.
2. This manuscript concentrates on demyelination, particularly in MS. It is important to summarize the current literature on mechanisms governing mature oligodendrocyte viability in MS and its animal models. Recent studies demonstrate the critical roles of PERK, NF-kB, among other signaling pathways in modulating oligodendrocyte viability and demyelination in MS and its animal models. These studies should be cited and discussed.

Is the review written in accessible language?

Yes

Are all factual statements correct and adequately supported by citations?

Yes

Are the conclusions drawn appropriate in the context of the current research literature?

Yes

Is the topic of the review discussed comprehensively in the context of the current literature?

Yes

Reviewer Expertise:

oligodendrocyte biology and pathology

I confirm that I have read this submission and believe that I have an appropriate level of expertise to confirm that it is of an acceptable scientific standard, however I have significant reservations, as outlined above.

# Response

**Published:** 2021-11-01

**Authors:** David Selwood

**Affiliations:** Department of Biological and Medicinal Chemistry, University College London, UK

We would like to thank Prof Lin for his careful reading of the manuscript and useful comments.

**Pt 1.** The reviewer asks if replacing “remyelination” with “demyelination” in the title would be more reflective of the review. The reviewer is correct in ascribing much of the review subject to demyelination, however, using “demyelination” in the title would make an association of demyelination with BK channels that would not be correct and we would not wish to do this. We think the modified title “ *Oligodendrocytes, BK channels and the preservation of myelin”* could be a better reflection of the subject and aims of the review.

**Pt 2. **It is important to consider other targets that may protect mature oligodendrocytes from demyelinating pathologies like multiple sclerosis. This is to establish whether BK channel activators could be a valid alternative or complement existing approaches.

 Another strategy could modulate the unfolded protein response (UPR) to preserve cell viability during periods of endoplasmic reticulum stress, like those caused by excessive inflammation, by temporarily halting translation to attempt re-establishing protein homeostasis. We will insert the text below into the final version of the manuscript.

*Pancreatic endoplasmic reticulum kinase (PERK) is implicated in this. Increasing PERK activation in mouse models of MS protected myelin, in the presence of MS- related cytokine interferon- γ (133). This even improved survival of remyelinating oligodendrocytes and recovery in demyelinated lesions (133). A later study indicated that the PERK protective pathway can be enhanced by nuclear factor κB (NFκB) activation (134). Oligodendrocytes were rescued only in mice with MOG- induced EAE and PERK gene deficiency, whilst healthy oligodendrocytes remained unaffected (134). This might indicate this UPR signalling pathway specifically protects from inflammatory and demyelinating pathology. Studies have replicated these cytoprotective effects, but also indicated the need for targeted activation of NFkB (135, 136, 134). Excessive generalised activation may worsen inflammation by dysregulating immunity and promoting autoimmune reactions, possibly leading to adverse effects (137).*

*Alternatively, the leukaemia inhibitory factor (LIF), of the IL-6 cytokine family, was also proposed to protect mature oligodendrocytes from demyelination. Its levels determine growth and differentiation of inflammatory cells, but also possibly neural cells. Exogenous LIF protected against demyelination in cuprizone induced mouse models of MS with knockout endogenous LIF (138). Since cuprizone is toxic to oligodendrocytes but does not elicit an inflammatory response as seen in the EAE model, the authors indicated this method is directly cytoprotective (138). However, some studies suggest its activation might lead to negative effects on neuroinflammation, and state limited ability to cross the BBB when administered therapeutically; although its delivery and effectiveness significantly improved when using lentiviral vectors for CNS- specific expression in murine models (139, 138).*

*Similarly, agents aimed at attenuating the inflammation were also discovered to have independent neuroprotective effects which can lead to reduced demyelination. For example repurposing the antibiotic minocycline, which is thought to enhance anti-apoptotic signalling and was combined with atorvastatin to achieve an enhanced reduction in EAE severity (140). In a randomised controlled trial, taking minocycline within the first 6 months of the clinically isolated syndrome significantly decreased the risk of developing MS but not after two years, which may suggest a delaying instead of disease- modifying effect (141).*

*Finally, in the MS lesion microenvironment, the excessive inflammation and failure of energy metabolism may lead to acidosis and subsequent activation of acid sensing ion channels (ASICs), which were found upregulated in both axons and oligodendrocytes in active MS lesions (142). These proton gated cation channels are activated in similar conditions of excitotoxicity as the BK channel. Amiloride or psalmotoxin-1, blocking respectively ASICs or ASIC1, protected oligodendrocytes from injury related to this acidosis, and the EAE mouse model with knockout ASIC1 had increased levels of myelin immunostaining compared to wild type (142).*

References:

**133.** Lin, Y., Huang, G., Jamison, S. & Li, J. et al. (2014) PERK Activation Preserves the Viability and        Function of Remyelinating Oligodendrocytes in Immune-Mediated Demyelinating Diseases. *The American Journal of Pathology*. [Online] 184 (2), 507-519. Available from: doi:10.1016/j.ajpath.2013.10.009.

**134. **Lei, Z., Yue, Y., Stone, S. & Wu, S. et al. (2020) NF-κB Activation Accounts for the Cytoprotective Effects of PERK Activation on Oligodendrocytes during EAE. *The Journal of Neuroscience*. [Online] 40 (33), 6444-6456. Available from: doi:10.1523/jneurosci.1156-20.2020.

**135. **Stone, S., Jamison, S., Yue, Y. & Durose, W. et al. (2017) NF-κB Activation Protects Oligodendrocytes against Inflammation. *The Journal of Neuroscience*. [Online] 37 (38), 9332-9344. Available from: doi:10.1523/jneurosci.1608-17.2017.

**136. **Tanner, D., Campbell, A., O'Banion, K. & Noble, M. et al. (2015) cFLIP is critical for oligodendrocyte protection from inflammation. *Cell Death & Differentiation*. [Online] 22 (9), 1489-1501. Available from: doi:10.1038/cdd.2014.237.

**137. **Ellrichmann, G., Thöne, J., Lee, D. & Rupec, R. et al. (2012) Constitutive activity of NF-kappa B in myeloid cells drives pathogenicity of monocytes and macrophages during autoimmune neuroinflammation. *Journal of Neuroinflammation*. [Online] 9 (1). Available from: doi:10.1186/1742-2094-9-15.

**138. **Marriott, M., Emery, B., Cate, H. & Binder, M. et al. (2008) Leukemia inhibitory factor signaling modulates both central nervous system demyelination and myelin repair. *Glia*. [Online] 56 (6), 686-698. Available from: doi:10.1002/glia.20646.

**139. **Slaets, H., Hendriks, J., Van den Haute, C. & Coun, F. et al. (2010) CNS-targeted LIF Expression Improves Therapeutic Efficacy and Limits Autoimmune-mediated Demyelination in a Model of Multiple Sclerosis. *Molecular Therapy*. [Online] 18 (4), 684-691. Available from: doi:10.1038/mt.2009.311.

**140. **Luccarini, I., Ballerini, C., Biagioli, T. & Biamonte, F. et al. (2008) Combined treatment with atorvastatin and minocycline suppresses severity of EAE. *Experimental Neurology*. [Online] 211 (1), 214-226. Available from: doi:10.1016/j.expneurol.2008.01.022.

**141. **Metz, L., Li, D., Traboulsee, A. & Duquette, P. et al. (2017) Trial of Minocycline in a Clinically Isolated Syndrome of Multiple Sclerosis. *New England Journal of Medicine*. [Online] 376 (22), 2122-2133. Available from: doi:10.1056/nejmoa1608889.

**142. **Vergo, S., Craner, M., Etzensperger, R. & Attfield, K. et al. (2011) Acid-sensing ion channel 1 is involved in both axonal injury and demyelination in multiple sclerosis and its animal model. *Brain*. [Online] 134 (2), 571-584. Available from: doi:10.1093/brain/awq337.

# Peer review: Reviewer response for version 1

**DOI:** 10.5256/f1000research.56803.r91531

**Published:** 2021-08-26

**Authors:** Enrique Balderas-Angeles, Veronica Loyo-Celis

**Affiliations:** 1 Nora Eccles Harrison Cardiovascular Research & Training Institute, University of Utah, Salt Lake City, UT, USA; 2 Departamento de Genética del Desarrollo y Fisiología Molecular, Instituto de Biotecnología Universidad Nacional Autónoma de México, Cuernavaca, Morelos, Mexico

**Is the topic of the review discussed comprehensively in the context of the current literature?**

 In the review by Rupnik *et al.,* the role of excitotoxicity in oligodendrocytes was approached through a physiological, clinical, and biophysical perspective, which allows the reader to understand the basics on the origin, progression, and possible therapies for diseases associated with demyelination.

**Are all factual statements correct and adequately supported by citations?**

 In the manuscript by Rupnik *et al.,* the statements were adequately supported by citations which included some classic works (Hursh, 1939 ^1^; Waxman *et al.*, 1977 ^2^; Waxman *et al.,* 1984 ^3^) and articles published in the last ten years.

**Is the review written in accessible language?**

 The review was written fluently, clearly and the topics were discussed deeply enough. The structure of the paper was clearly defined at the beginning of the text (page 3).

**Are the conclusions drawn appropriate in the context of the current research literature?**

 The authors summarized the causes and effects of oligodendrocytes toxicity and proposed an alternative therapy to treat pathologies associated with demyelination. One strategy involves modulation of the large conductance, Ca ^2+^ and voltage-activated BKCa channel, which activation might prevent Ca ^2+^ overload to protect oligodendrocytes from excitotoxic stress. Of note,  a similar mechanism has been observed in cardiac mitochondria in rodents, where the BKCa channel expresses and its activation reduces the driving force for Ca ^2+^, preventing overload and cell death (Singh *et al.,* 2013 ^4^; Balderas *et al.,* 2019 ^5^). Moreover, as observed in cardiac mitochondria, the functional association of mitoBK _Ca_ with regulatory subunits β1 broadens the spectrum of molecular targets suitable for the development of new and more directed therapies.  

 Targeting the pore forming BKCa channel α-subunit with current and novel drugs needs to overcome the broad localization of the channel in brain cells. Considering that BKCa is expressed ubiquitously in brain cells it is essential to establish the differential and perhaps localized expression of BKCa channels and/or its regulatory subunits (β1-4; γ1-4) in oligodendrocytes. This would allow designing a more specific therapy alternative to immunosuppressive agents.

 In summary, the review presented by Rupnik and coworkers represents a good collection of most recent information in the field of oligodendrocytes and the pathologies associated with demyelination, highlighting a possible role of the BKCa channel in preventing Ca ^2+^ overload and reducing excitotoxic stress. 

 Our main suggestion would be to include a comment on the importance of the expression and association with BKCa-regulatory subunits and their possible use as novel targets for future therapies.

Is the review written in accessible language?

Yes

Are all factual statements correct and adequately supported by citations?

Yes

Are the conclusions drawn appropriate in the context of the current research literature?

Yes

Is the topic of the review discussed comprehensively in the context of the current literature?

Yes

Reviewer Expertise:

MaxiK, BK, mitochondrial ion channels, cardiac disorders, cardiovascular disease, vascular tone, immunotherapy, Ca2+ handling, ROS, cellular volume, cell death.

We confirm that we have read this submission and believe that we have an appropriate level of expertise to confirm that it is of an acceptable scientific standard.

## References

[1] : CONDUCTION VELOCITY AND DIAMETER OF NERVE FIBERS. *American Journal of Physiology-Legacy Content* .1939;127(1) : 10.1152/ajplegacy.1939.127.1.131 131-139 10.1152/ajplegacy.1939.127.1.131

[2] : The conduction properties of axons in central white matter. *Progress in Neurobiology* .1977;8: 10.1016/0301-0082(77)90009-0 297-324 10.1016/0301-0082(77)90009-0 335441

[3] : Specificity in central myelination: evidence for local regulation of myelin thickness. *Brain Research* .1984;292(1) : 10.1016/0006-8993(84)90905-3 179-185 10.1016/0006-8993(84)90905-3 6697207

[4] : MitoBK(Ca) is encoded by the Kcnma1 gene, and a splicing sequence defines its mitochondrial location. *Proc Natl Acad Sci U S A* .2013;110(26) : 10.1073/pnas.1302028110 10836-41 10.1073/pnas.1302028110 23754429PMC3696804

[5] : MitoBKCa channel is functionally associated with its regulatory β1 subunit in cardiac mitochondria. *J Physiol* .597(15) : 10.1113/JP277769 3817-3832 10.1113/JP277769 31173379PMC6690848

# Response

**Published:** 2021-10-13

**Authors:** David Selwood

**Affiliations:** Department of Biological and Medicinal Chemistry, University College London, UK

Response to Enrique Balderas-Angeles, and Veronica Loyo-Celis,

 We thank the reviewers for their careful reading of the manuscript and useful comments and respond below.  A new paragraph expressing some of these points will be added to the final manuscript.

 Based on the reviewed evidence, there is an association of BK channel subunits with disease. BK channel activation is neuroprotective in animal models of spinal cord injury, ischaemia, and excitotoxic stress (50, 113, 115, 125), which share some pathological similarities with MS. This is confirmed by allele knockout or blockage with the antagonist's iberiotoxin and/ or paxilline that exacerbated injury (113, 115). The agonist isopimaric acid targeted the α subunit to improve motor function in rats with spinal cord injury (50). Similarly, NS1619 activated subunit α1 to effectively increase neuroprotection in rat and mouse cortex when used with leptin (115). Certain subunits may only be activated and therefore effectively targeted in the presence of cellular injury or excitotoxic conditions (114, 125), a quality which could arguably improve the specificity of an agent to injured tissue. However, these studies focused on neuron expression of BK channels and axon rather than oligodendrocyte integrity as an outcome. In clinical trials, BMS-204352 activates KCNMA1 also activates another potassium channel KCNQ. This was trialed as a safer neuroprotective agent to reduce intracellular Ca ^2+^ levels in acute ischaemic stroke but did not significantly improve the outcome in this study (128). Andolast has been shown to significantly improve asthma symptoms compared to placebo, but its subunit targets have not yet been investigated (129). The anti-glaucoma agent unoprostone isopropyl activated iberiotoxin resistant BK channels, therefore likely successfully targeted the β2, or β3, subunits (130). The β regulatory subunit is generally quite tissue-specific and therefore arguably a better target for an agonist than the α- subunit (121). In the CNS, KCNMB4 and KCNMB2 are the main regulatory subunits expressed (131). VSN16R, which is thought to target preferentially the β4 subunit, has shown promise in reducing spasticity at higher doses with few side effects (131, 132). Notably, KCNMB4 is expressed in human mature oligodendrocytes (figure 3) and is possibly more abundant in chronic inactive MS lesions (117). Evidence is needed to ascertain whether an agonist to KCNMB4, or to another regulatory subunit, changes outcome in MS pathogenesis by targeting oligodendrocytes and by preserving myelin. Recently Fragile X syndrome the major genetic cause of intellectual disability has been shown to have a demyelinating component. BK channel activation (specifically alpha1beta4) has been shown to restore both electrophysiological and behavioural functions in experimental Fragile X. A clinical evaluation should examine if remyelination is a component of the response to BK activator therapy.
